# Supplementary material for: Time-resolved imaging of pulse-induced magnetization reversal with a microwave assist field
Source: Sci Rep. 2015 May 29;5:10695. doi: 10.1038/srep10695 (PMC4448658; doi:10.1038/srep10695)
Supplement: Supporting Information [file srep10695-s1.pdf]

Time-resolved imaging of pulse-induced magnetization reversal with a microwave assist field

Siddharth Rao,[1] Jan Rhensius,[1] Andre Bisig,[2, 3] Mohamad-Assaad Mawass,[2, 3] Markus Weigand,[3] Mathias Kläui,[2] Charanjit S. Bhatia[1] & Hyunsoo Yang[1]

[1]*Department of Electrical and Computer Engineering, National University of Singapore, 4 Engineering Drive 3, Singapore 117576, Singapore*

[2]*Institute of Physics, University of Mainz, Staudinger Weg 7, 55128 Mainz, Germany*

[3]*Max-Planck-Insitut fur Intelligente Systeme, Heisenbergstr. 3, 70569 Stuttgart, Germany*

1. Spatial variation of demagnetizing field

The demagnetizing field across the 4 × 0.4 μm^2 element is determined by simulations. The element is saturated along its easy axis in the initial state and allowed to relax for 5 ns. Figures S1(a-c) show the components of the demagnetizing field in the *x*, *y*, and *z*-directions. In particular, the field is weak near the foci of the element in the *x* and *y* directions, and very strong in the *z* direction. This suggests that the magnetization reversal is an in-plane process.

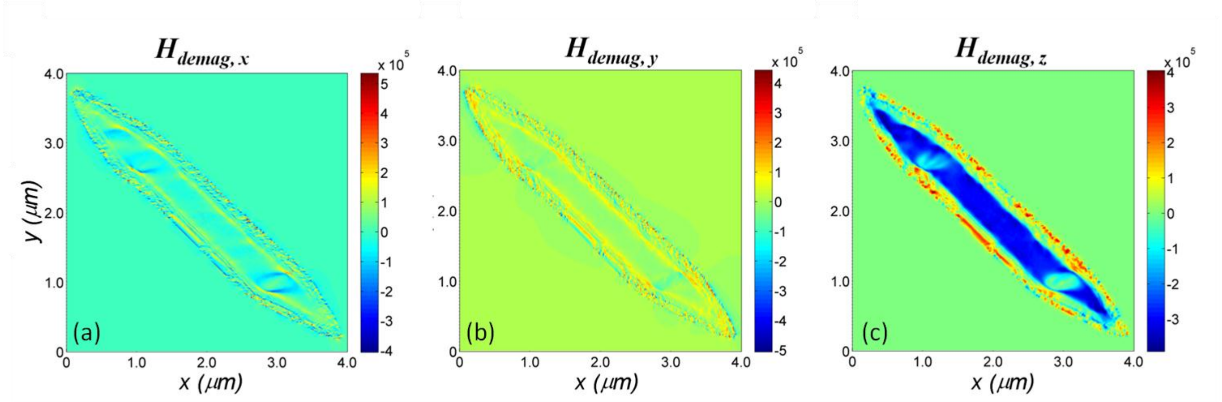

FIG. S1. Spatial variation of the demagnetizing field across the elliptical element. Components of the demagnetizing field along the *x*-direction (a), *y*-direction (b), and *z*-direction (c).
